# Effect of elastic constants on electrically induced transition in twisted radial cholesteric droplets

**DOI:** 10.1038/s41598-022-13612-4

**Published:** 2022-06-10

**Authors:** Vladimir Yu. Rudyak, Mikhail N. Krakhalev, Anna P. Gardymova, Abylgazy S. Abdullaev, Andrey A. Alekseev, Victor Ya. Zyryanov

**Affiliations:** 1 Faculty of Physics, Lomonosov Moscow State University, Moscow, Russia 119991; 2 Kirensky Institute of Physics, Federal Research Center KSC SB RAS, Krasnoyarsk, Russia 660036; 3 Institute of Engineering Physics and Radio Electronics, Siberian Federal University, Krasnoyarsk, Russia 660041

**Keywords:** Phase transitions and critical phenomena, Liquid crystals, Polymers, Sensors and biosensors

## Abstract

In this work, we investigated the behavior of cholesteric droplets with homeotropic boundary conditions experimentally and by computer simulations. Small droplets forming twisted radial structures were studied. We obtained two different paths of structural transformations under electric field in such droplets. The choice between these paths has probabilistic nature. The ratio between the two transition types was found to be sensitive to the elastic constants of LC forming the droplet. We suggest the principal approach for *in situ* estimation of ratios between elastic constants in cholesteric LCs deposited in polymer-dispersed LC material and discuss its strong and weak sides.

## Introduction

The study of liquid crystals confined to microscopic cavities demonstrates growing interest for the last decades, both from the fundamental point of view^1–5^ and applications^6–10^, including sensors^11–13^, display technologies^14^, biological applications^15,16^ and other^17–19^. The direct connection between topological defects in particle physics and liquid crystals allows using liquid crystals as convenient systems for studies of several fundamental problems^20–22^. Last decade, the most intriguing discoveries shift from nematic to cholesteric liquid crystals (CLC). CLCs demonstrate plenty of various director configurations in confined geometries. For example, twisted bipolar structure^13,23^, structures with diametrical $\chi ^{+1}$ or radial $\chi ^{+2}$ dislocations^23,24^, uniform helix axis distribution^25^ and the double twisted the structure^26^ can be observed, as well as structures with the point defect in the bulk of CLC or at the surface and bipolar distribution of CLC axes^27,28^.

**Figure 1:**
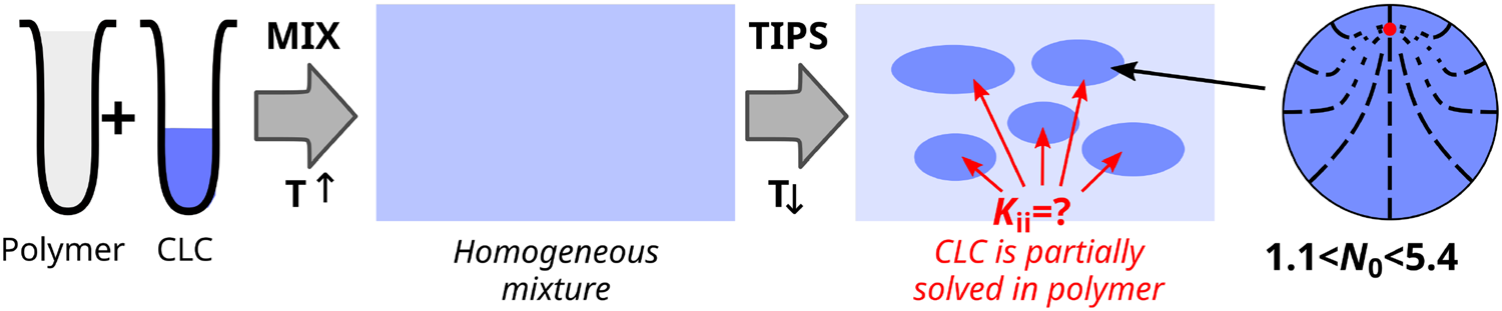
Principal scheme of preparation of polymer dispersed liquid crystalline material. After temperature-induced phase separation (TIPS), part of a liquid crystal remains solved in polymer, affecting the mixture contained in a droplet

One of the practically important ways to fabricate, examine and use LCs in confined geometries are polymer-dispersed liquid crystal (PDLC) films. Typically, it is prepared by phase separation methods by mixing the polymer with LC in a homogeneous, single-phase blend. The subsequent phase separation can be induced in several ways. In temperature-induced phase separation (TIPS), a melted mixture of thermoplastic polymer and LC is placed on a substrate and gradually cooled down until polymer solidification induces phase separation (Fig. 1). In the end, LC becomes only partially soluble in a polymer matrix and form small LC droplets inside the film. While this approach has massive benefits like the ability to prepare material en masse and thermomechanical stability of the material, there are also payoffs. Most applications require using specific LC mixtures instead of pure LC compounds. Solubility rates of different parts of these compounds may significantly vary^6,29–32^. In turn, the content of an LC mixture in a PDLC droplet may differ from the original LC mixture due to various adsorption rates to the matrix of different components of a mix. And finally, it will affect the material properties of the LC mixture within the droplet (for example, elastic constants), its optical properties, and response to an electric field^33,34^. Thus it is essential to estimate the properties of LC material inside PDLC droplets after film preparation.

There are seemingly two ways to solve the problem of estimating an LC mixture content in PDLC droplets. First, one can study adsorption rates and then evaluate the mixture composition. As there are many different LC compounds and polymer matrices, it is typically done theoretically^35,36^. Second, it is possible to measure optical structural features of the end droplets by means of microscopy and estimate material properties from it. This is an empirical approach requiring more complex studies. On the other side, its important benefit is that it can be based on experimental observations or directly compared with it. Recently this problem was addressed^37^ by simulating polarized microscopy images of a large set of structures for various elastic constants.

In this work, we studied the behavior of cholesteric droplets with homeotropic boundary conditions experimentally and by computer simulations. Small droplets forming twisted radial structures were studied. We obtained two different paths of structural transformations under electric fields in such droplets. The choice between these paths has probabilistic nature. The ratio between the two transition types was found to be sensitive to the elastic constants of LC forming the droplet. Thus we suggest the principal method for *in situ* estimation of ratios between elastic constants in cholesteric LCs deposited in PDLC material.

## Results

**Figure 2:**
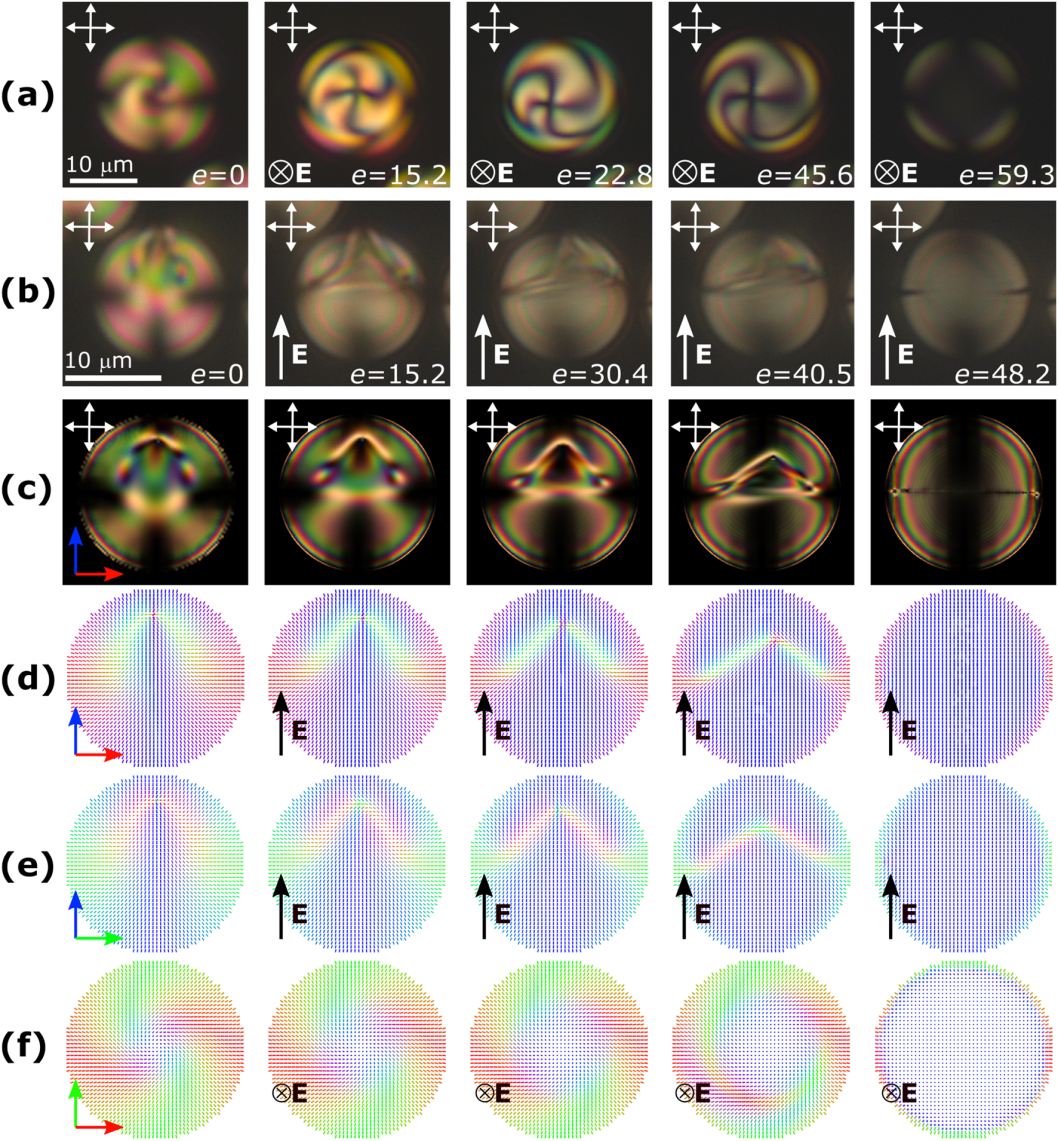
Sequence of structures for the symmetric transition scenario in increasing electric field $e=|{\mathbf {E}}|d(\varepsilon _0\Delta \varepsilon /4K_{11})^{1/2}$. Experimental cross-polarized microscopy images for (**a**) symmetry axis perpendicular to the plane, $N_0=2.2$, and (**b**) symmetry axis parallel to the plane, $N_0=1.8$. Simulated cross-polarized images (**c**) and director distribution in primary cross cuts (**d**–**f**) for E7, $N_0=2.5$. Hereinafter, double arrows indicate the direction of the polarizers, and the director ${\mathbf {n}}$ is colored in correspondence with the direction (red along the *x*-axis, green along the *y*-axis, blue along the *z*-axis).

PiBMA polymer assigns the homeotropic anchoring for the E7 nematic^38^. It is known that twisted radial defect (**tR**) structure with a single point defect shifted to the droplet edge is formed in the range of $N_0=2d/p_0$ between 1.1 and 5.4, where *d* is droplet diameter and $p_0$ is equilibrium cholesteric pitch^28^. At $N_0 = 2.4$ ($d = 20\,\mu$m) this defect lies almost at the border (see Figs. 1 and  2, left column). Simultaneously, the high overall twist of the director field compensates for the twisting power of the CLC, which is visualized by the extinction lines of the radial structure. In simulations, this structure was fully confirmed in the range $1.3<N_0<3.2$.

### Transition dynamics under electric field

We studied the behavior of droplets with **tR** structure under the action of an electric field. In both experiment and computer simulations, two possible sequences of structure transformation were obtained. In one case, the droplet remained axially symmetrical all the time; in another, the symmetry broke and was then restored only after transition to toron configuration. Below we depict both cases in detail.

Fig. 2 shows the sequence of symmetric droplet configurations in increasing electric field (hereafter the value of electric field is shown in dimensionless form $e=|{\mathbf {E}}|d(\varepsilon _0\Delta \varepsilon /4K_{11})^{1/2}$, see details in Methods section). In Fig. 2 rows (a) and (b) we show experimental results for the droplets of $d=18\mu$m ($N_0=2.2$) and $d=15\mu$m ($N_0=1.8$), correspondingly. The structures in these droplets are very similar but oriented by 90$^\circ$ to each other, as well as the electric field. The same transition in computer simulations is shown in rows (**c**)-(**f**) for a droplet with $N_0=2.5$. In all cases, in a low electric field, **tR** structure symmetry axis is parallel to the electric field, and point defect shifts close to the border of the droplet. In an intermediate electric field, the point defect slowly shifts toward the center of the droplet. Simultaneously, the twisted cone becomes thinner, which significantly changes its visual appearance in cross-polarized microscopy. Finally, the point defect breaks down into a circular line defect, and the droplet rapidly transforms into the toroidal configuration in a high electric field (Fig. 2, right column).

**Figure 3:**
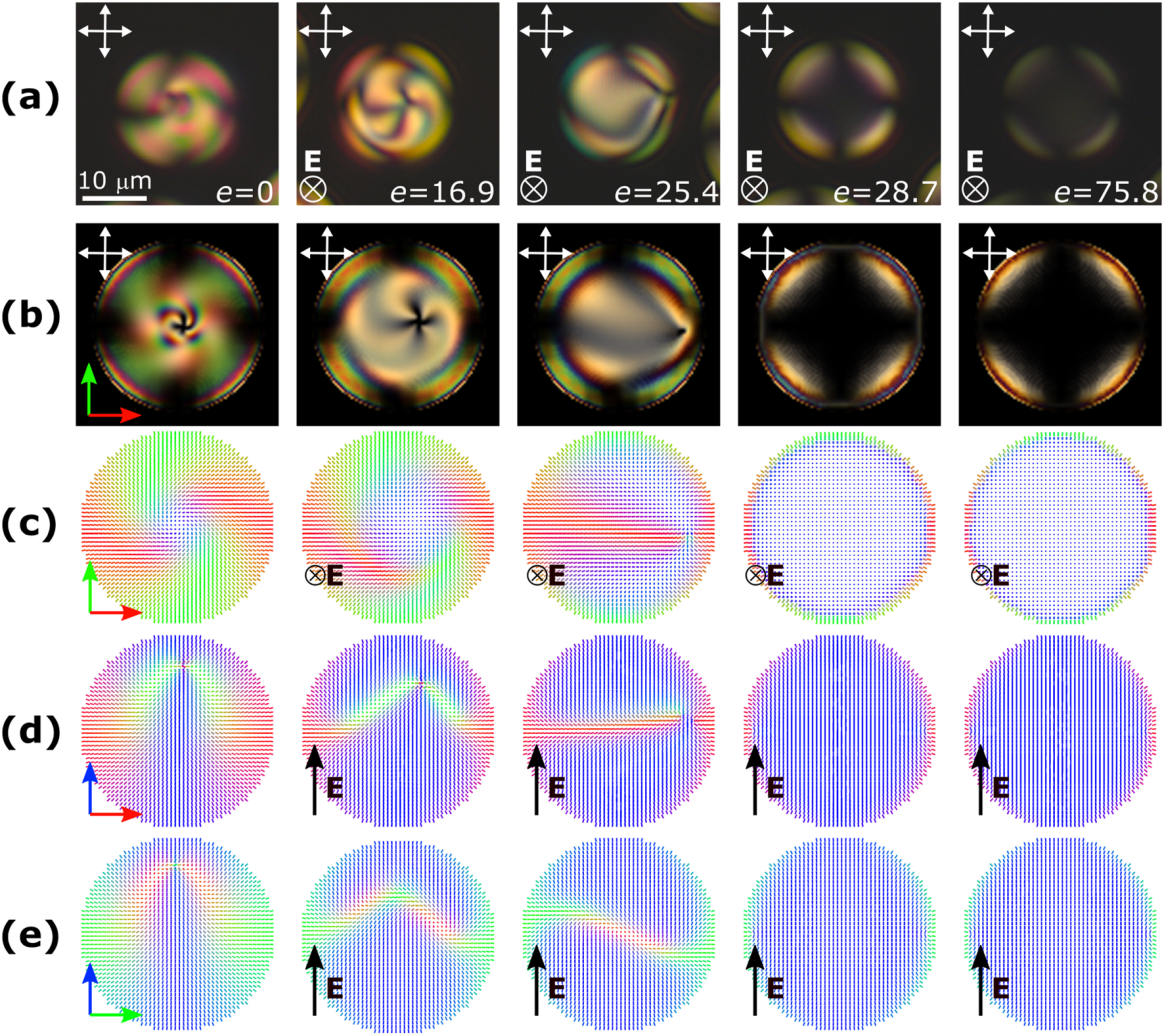
Sequence of structures for the asymmetric transition scenario in increasing electric field. Experimental cross-polarized microscopy images, $N_0=2.4$ (**a**), and simulated cross-polarized images (**b**) and director distribution in primary cross cuts (**c**–**e**) for E7, $N_0=2.5$.

Another possible scenario is shown in Fig. 3. Here, the experiment starts from the same initial **tR** structure in the absence of an electric field (left column). However, at an intermediate electric field, the point defect rapidly shifts to the side of the droplet, and the structure loses its symmetry. In further increasing electric field, the point defect approaches the border of the droplet, and the droplet structure undergoes reorientation (Fig. 3, third column). Alongside with strongly pronounced asymmetry, the character of the director field deformations around the defect changes significantly. Instead of symmetrical zone of twist nearby the defect in symmetrical case, here the two-wing structure with pronounced splay and low bend deformation occurs (see Supplementary Fig. S1). These changes are clearly seen in both cross-polarized microscopy images and director distributions. Accordingly, splay energy increases during symmetrical-to-asymmetrical transition, bend energy decreases, and twist enegry remains nearly constant. Finally, in a high electric field, the transition into toroidal configuration occurs (Fig. 3, fourth column). In the asymmetric scenario, it happens at a much lower *e*, than in symmetric case, regardless of droplet size.

### Probabilities of symmetric and asymmetric transitions

In experiment and computer simulations, we obtained transitions through both scenarios in droplets of all sizes in the range of $N_0$ from $\sim 1.5$ to $\sim 4$. In the experiment, we were unable to determine any specific feature in a droplet at $e=0$, that would have determined the case. In simulations, a repeat of exactly the same state and simulation protocol led to one symmetric transition in some trials and asymmetric one in others. Thus the particular choice of transition scenario for every single droplet was seemingly probabilistic.

**Figure 4:**
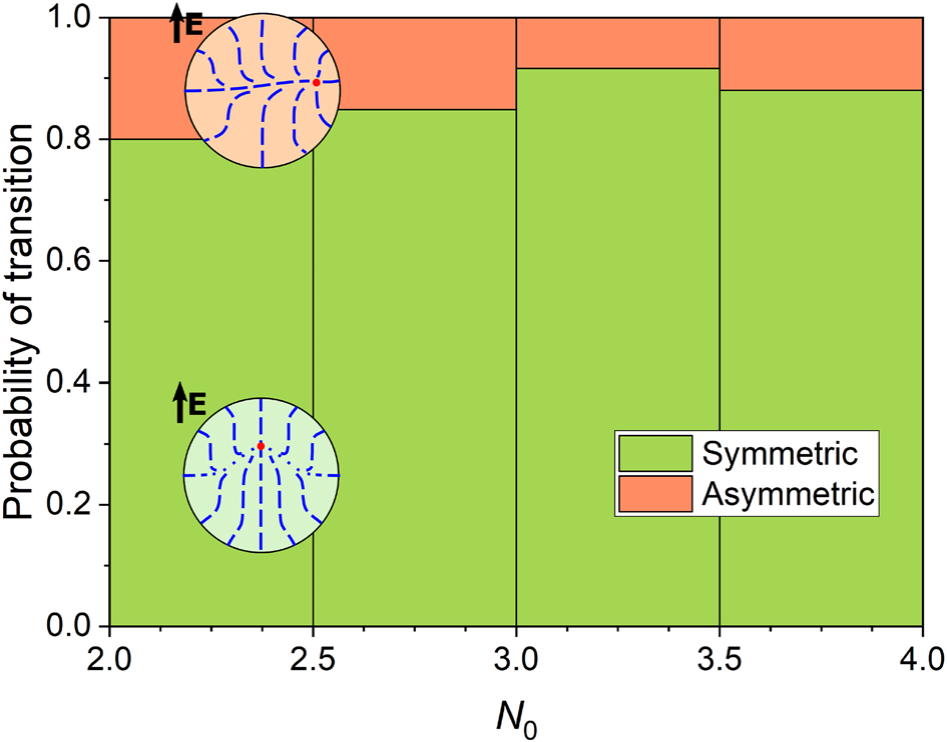
Probabilities of symmetric (green) and asymmetric (red) transitions at various relative droplet helicity parameters $N_0$, experimentally measured for 745 E7 droplets in PiBMA.

To quantify the experimental data, we analyzed transition type in 725 droplets with $N_0$ from $2.25 \pm 0.25$ to $3.75 \pm 0.25$. Only those droplets were taken into account, the symmetry axes of which were initially oriented mainly along the applied electric field. We considered the structural transition in a droplet as the symmetric scenario if the point defect shifted from the center less than half of the droplet radius in the whole range of *e* before transition to the toroidal configuration. Otherwise, the case was counted as the asymmetric scenario. Then we split up all data by droplet size and calculated the probabilities of the two scenarios in each group (Fig. 4). On average, the probability of the asymmetric scenario was approximately 14% and showed only a slight dependency on droplet size.

**Figure 5:**
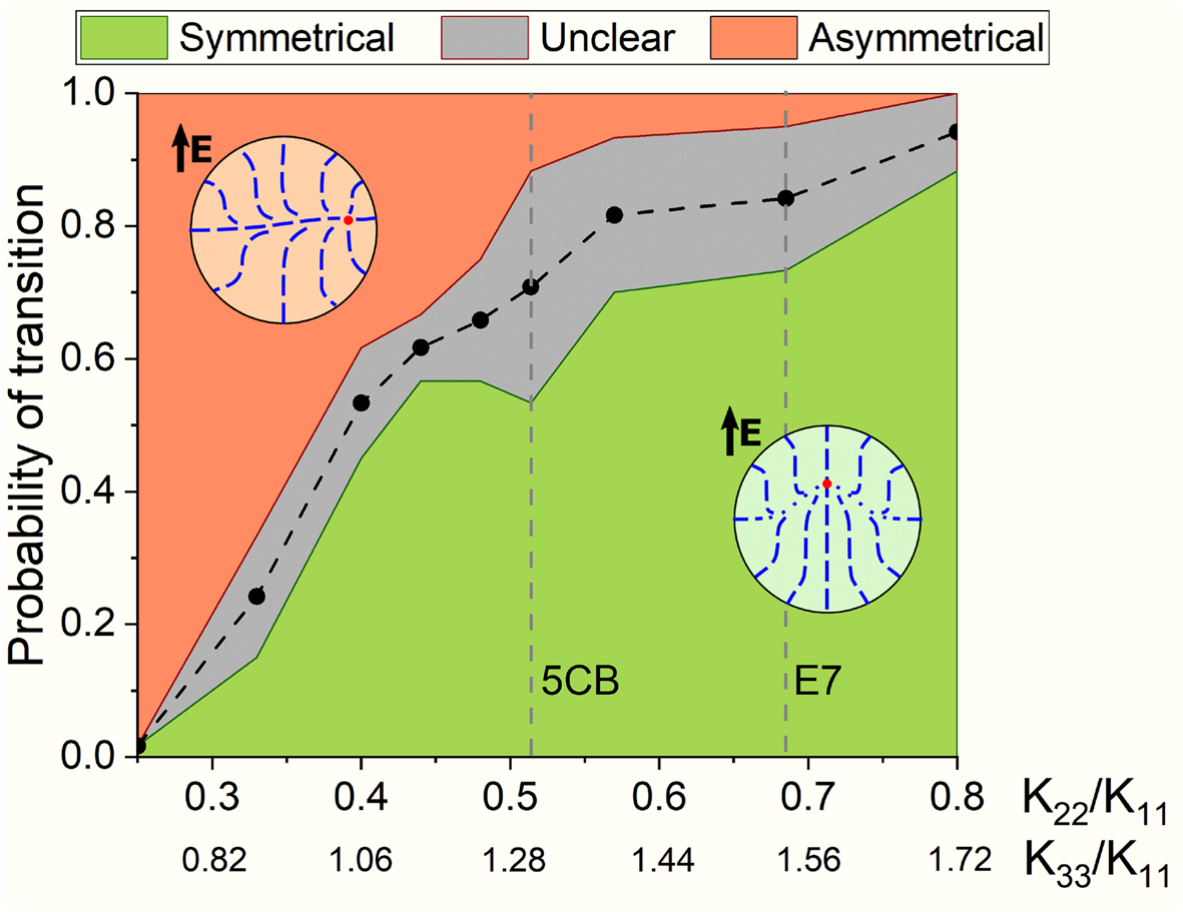
Probabilities of symmetric (green), asymmetric (red), and unclear (grey) transitions at relative droplet helicity parameter $N_0=2.5$ and various elastic constants, calculated from 150 computer simulation runs per point. The black dashed line shows the middle of the “unclear” zone, which estimates experimentally observable data.

For theoretical analysis, we produced simulations of transition in $N_0=2.5$ droplets under the electric field for materials with various ratios between elastic constants $K_{11}:K_{22}:K_{33}$. To obtain more realistic materials in a single row of gradually changing parameters, including E7 and 5CB liquid crystals, we changed both $K_{22}$ and $K_{33}$ simultaneously. In each case, we produced 150 independent simulations (total of 1350 runs) and split the results into three groups: symmetric, asymmetric, and unclear scenarios. The latter was assigned when the structure fluctuated significantly before the transition to toroidal configuration. Figure 5 shows the resulting probability distribution. First, simulation results for E7 are very close to the experimental data. Second, the obtained distribution between probabilities of symmetric and asymmetric scenarios strongly depends on the elastic constants. Low $K_{22}/K_{11}$ appeared to be the most sensitive parts, with the number of symmetric cases rising from almost zero at $K_{22}/K_{11}=0.25$ to nearly two thirds for 5CB. The changes between 5CB and E7 and further are less significant but still reasonably detectable. Additionally, to verify theoretically the impact of the the droplet size on the transition probability, we produced 60 independent simulations with $N_0$ equal to 3.0 and 3.5 for E7 LC material. The probabilities of symmetrical, unclear and asymmetrical scenarios were 73%:10%:17% and 74%:13%:13% for $N_0=3.0$ and 3.5, correspondingly. Thus, the estimated symmetrical transition probabilities for $N_0$ equal to 2.5, 3.0 and 3.5 are 84%, 78% and 80%, correspondingly.

## Conclusions and discussion

The response of the twisted radial structure formed in cholesteric droplets at $1.5< N_0 < 4.0$ to an applied electric field has been studied in detail. This structure is axially symmetrical and characterized by the bulk point defect located near the droplet boundary. If the symmetry axis of the structure is initially oriented parallel to the applied electric field, then two scenarios of transformation are observed at increasing voltage. In the first scenario, the structure symmetry remains, the cone-shaped region where the director is oriented perpendicular to the electric field appears. The cone vertex coincides with the point defect, and its axis is parallel to the electric field. In the second scenario, the axial symmetry of the structure is broken and the point defect shifts along the droplet edge into the droplet equatorial plane perpendicular to the applied electric field. In this equatorial plane appears the sector where the director is oriented perpendicular to the applied field. The structures with the point defect transition into a toroidal configuration with a surface ring defect in a high electric field. The dimensionless field e at which this transition occurs is smaller with the asymmetric transformation scenario for droplets of cholesteric based on nematic E7.

The ratio between the two transformation scenarios was shown to be highly sensitive to the LC elastic constants ratio $K_{11}:K_{22}:K_{33}$. For instance, probability of the structure symmetrical transition increases from about 0 to 0.9 at increasing $K_{11}:K_{22}:K_{33}$ ratio from 1:0.25:0.69 to 1:0.8:1.72. We believe this effect potentially allows us to determine *in situ* the ratio between elastic constants in cholesteric LC mixtures inside the PDLC matrix. In contrast to the approach based on the POM at absence of electric field^37^, our method is based on the analysis of system response to the external electric field. As a result, it is more sensitive to the changes in elastic constants. Thus, on one side, it can be used only in a small range of elastic constant ratios. On other side, it allows to distinguish materials with relatively close elastic properties (for example, 5CB and E7). We consider the following blueprint of the protocol for such measurements: (1) estimate oblateness of the droplets under study; (2) estimate the possible range of values for the elastic constant ratios; (3) measure symmetric and asymmetric transition probabilities and check reference tables for the specific polymer (these data are to be collected in future studies).

We understand that the applied measurements require more detailed research and testing. First of all, more extensive studies in a broad range of elastic constant ratios are needed. Ideally, it should be done for pure LCs or LC mixtures with a known matrix absorption ratio. We understand it is very hard to implement for a single matrix type. Thus we suggest composing the complete reference datasets by combining experiments and simulations. Second, the influence of droplet oblateness on the transition probabilities may be also considered. While our approach utilizes small droplets ($N_0<3-4$), which are almost spherical, some materials may result in oblate droplets. Previous data on droplet oblateness^39^ allows one to estimate it in a non-destructive manner. Third, it is unclear how to compare LC droplets in different matrixes. Different polymer materials may lead to varying strengths of surface anchoring, droplet shape, etc. Finally, the resulting numbers will strongly depend on the protocol in both experiments and simulations (for example, on the electric field growth rate). The data taken under different protocols can be compared only qualitatively but not quantitatively. An actual protocol can be adjusted to meet the technological requirements. As a primary option for laboratory measurements, we suggest the protocol used in this work. Despite these caveats, we believe the emphasized difficulties are vincible, as it requires more data but does not spoil the found effect. Thus we hope it can be used for *in situ* estimation of ratios of elastic constants in cholesteric LCs in the future.

## Methods

### Experimental

Experimental studies of the CLC droplet structures reaction were performed using polymer dispersed liquid crystal (PDLC) films. PDLC films were based on poly(isobutyl methacrylate) (PiBMA) (Sigma Aldrich) and the nematic mixture E7 (Merck) doped with cholesteryl acetate (Sigma Aldrich). To study the effect of the electric field on the cholesteric structures, it was used LC with the cholesteryl acetate concentration equal to 1 wt.% corresponding to the intrinsic helix pitch $p_0 = 16.4~\mu$m. To study the structural transitions statistics, it was used LC with the cholesteryl acetate concentration equal to 1.5 wt.% corresponding to the intrinsic helix pitch $p_0 = 10.9~\mu$m. The electro-optical sandwich-like cells were manufactured by combined phase separation technology as described in ref.^39^ in detail. The manufacturing conditions were chosen such as to obtain droplets with a relative chiral parameter in the range $1.3< N_0 < 4.5$. Two types of electro-optical cells were used, in which the electric field was applied *i)* perpendicular or *ii)* parallel to the plane of the PDLC film. Two identical ITO coated glass substrates were used to make electro-optical cells of the first type. In electro-optical cell of the second type, two ITO electrodes separated by $H \cong 100~\mu$m gap were on the one substrate, and the second glass substrate was without electrodes. The investigated PDLC film was placed between the substrates, $20~{\mu }$m diameter glass microspheres (Duke Scientific Corporation) were used for setting the composite film thickness. Experimental studies were carried out using a polarizing optical microscope (POM) Axio Imager.A1m (Carl Zeiss). AC electric field with 1 kHz frequency was applied to the electro-optical cells. The voltage amplitude was increased with the 1 V RMS step. The time between successive voltage increases was 60 sec.

### Computer simulations

#### Structure calculations

We performed simulations of the LC structure within spherical droplets with homeotropic boundaries filled with chiral nematic. The volume was rendered in $48\times 48\times 48$ lattices. We used extended Frank elastic continuum approach with Monte-Carlo annealing optimization^40^ to find the energy-optimal droplet structures. This approach includes the effects of the director field distortion and the formation of defects in the droplet:

1 $$\begin{aligned} \begin{aligned} F&=\int _V\left( \frac{K_{11}}{2}(\mathrm {div}{\mathbf {n}})^2 +\frac{K_{22}}{2}({\mathbf {n}}\cdot \mathrm {rot}{\mathbf {n}}+q_0)^2 +\frac{K_{33}}{2}\left[ {\mathbf {n}}\times \mathrm {rot}{\mathbf {n}}\right] ^2 \right) dV+\frac{W}{2}\int _\Omega \left( 1-\cos ^2\gamma \right) d\Omega \\&\quad +F_{def}+\varepsilon _0\Delta \varepsilon \int _V\left( {\mathbf {E}}^2-({\mathbf {E}}\cdot {\mathbf {n}})^2\right) dV, \end{aligned} \end{aligned}$$

where $K_{11}$, $K_{22}$ and $K_{33}$ are the splay, twist and bend elasticity constants, respectively, $q_0 = 2\pi /p_0$, *W* is the surface anchoring energy density, $\gamma$ is the angle between local director and normal to the droplet surface, $F_{def}$ is the energy of defects calculated by the summation of the point and linear defect energies, and $\Delta \varepsilon$ is dielectric anisotropy of LC, ${\mathbf {E}}$ is the electric field. The types, positions and energies of defects were estimated automatically during the Monte-Carlo optimization procedure (see the details in ref.^40^).

The initial structures in absence of an electric field, including the director field, the types, positions, and energies of defects were calculated during the Monte-Carlo optimization procedure (see the details in ref.^40^). To take into account potential formation of the disclination lines with core, its linear energy density was set to $f_{core}^{line} = 2.75 K_{11}$ (same as in^28^). The relative chiral parameter $N_0$ has been varied from 2.0 to 3.5. The homeotropic anchoring strength was set to strong, $\mu = Wd/2K_{11}$ varied from 1100 (for $N_0=2.0$) to 1950 (for $N_0=3.5$) to maintain constant natural units value of $W=1.75\times 10^{-3}$ J/$\hbox {m}^2$. To simulate changes in the structure under a slowly increasing electric field, we increased the amplitude of the electric field in small steps ($\Delta e=0.25$) and applied Monte Carlo relaxation at each step ($10^5$ Monte Carlo multisteps). The data are shown in dimensionless electric field, which were calculated as $e=|{\mathbf {E}}|d\left( \varepsilon _0\Delta \varepsilon /4K_{11}\right) ^{1/2}$.

#### Calculation of the POM textures

We have calculated the POM textures of spherical-cap droplets using the Jones matrix technique, formulated for PDLC materials in ref.^41^. This technique supposes direct unidirectional propagation of linearly polarized light through a non-uniform birefringent material. Light diffraction, diffusion, and scattering are not taken into account in the Jones calculus, and thus textures on the peripheral parts of the droplets are roughly estimated. Textures were calculated for ten different wavelengths within the visible spectrum, from 400 nm to 700 nm with the equal step of 33 nm. The values of ordinary and extraordinary reflective indices for E7 cholesteric were set in according to ref.^42^. Color textures were created by merging the individual wavelength textures with regard to the luminescence spectra of the black body at a temperature $\approx 3000 K$.

## Supplementary Information

Supplementary Information.

## Data Availability

The datasets generated during and analysed during the current study are available from the corresponding author on reasonable request.
