# Supplementary material for: Effect of elastic constants on electrically induced transition in twisted radial cholesteric droplets
Source: Sci Rep. 2022 Jun 10;12:9565. doi: 10.1038/s41598-022-13612-4 (PMC9187752; doi:10.1038/s41598-022-13612-4)
Supplement: Supplementary file 1 — Supplementary Information. [file 41598_2022_13612_MOESM1_ESM.pdf]

Supplementary Information
Effect of elastic constants on electrically induced transition in twisted radial cholesteric droplets

Vladimir Yu. Rudyak[1,*], Mikhail N. Krakhalev[2,3], Anna P. Gardymova[3], Abylgazy S. Abdullaev[2,3], Andrey A. Alekseev[1], and Victor Ya. Zyryanov[2]

[1]Faculty of Physics, Lomonosov Moscow State University, 119991 Moscow, Russia
[2]Kirensky Institute of Physics, Federal Research Center KSC SB RAS, 660036 Krasnoyarsk, Russia
[3]Institute of Engineering Physics and Radio Electronics, Siberian Federal University, 660041 Krasnoyarsk, Russia
[*]vurdizm@gmail.com

Comparison of energy terms and structure symmetry at the symmetrical-asymmetrical transition.

To distinguish symmetrical and asymmetrical states in computer simulations, we supported visual analysis with the aid of structure symmetry analysis. Fig. S 1 shows an example of evolution of splay and bend energies as well as a degree of structure asymmetry regarding z axis (axial symmetry will lead to zero degree of asymmetry). Red dashed line shows the transition point from symmetrical to asymmetrical structure. Symmetrical (green) and asymmetrical (black) scenarios are clearly distinguished by both asymmetry ratio and behavior of energy terms.

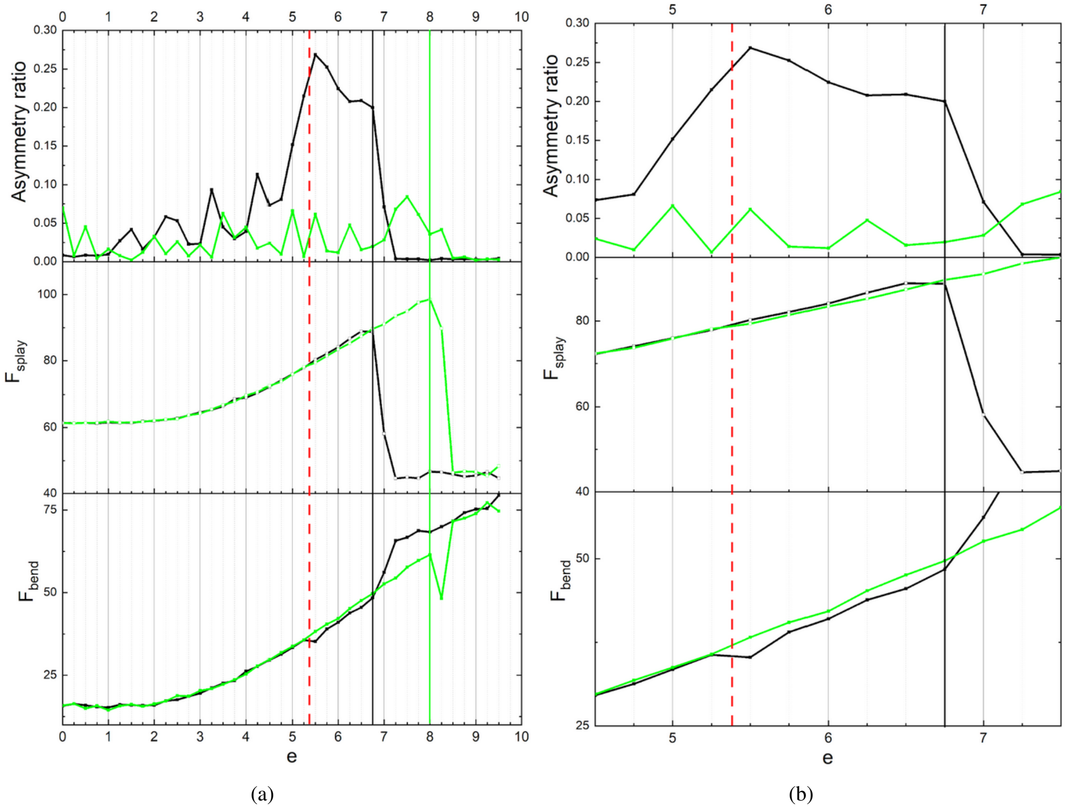

Figure S 1. Evolution of splay and bend energies as well as a degree of structure asymmetry (regarding z axis) in symmetrical (green) and asymmetrical (black) scenarios. Red dashed line shows the transition point from symmetrical to asymmetrical structure.
